# Recombinant attenuated *Salmonella* Typhimurium with heterologous expression of the *Salmonella* Choleraesuis O-polysaccharide: high immunogenicity and protection

**DOI:** 10.1038/s41598-017-07689-5

**Published:** 2017-07-28

**Authors:** Xinxin Zhao, Qinlong Dai, Dekang Zhu, Mafeng Liu, Shun Chen, Kunfeng Sun, Qiao Yang, Ying Wu, Qingke Kong, Renyong Jia

**Affiliations:** 1 Research Center of Avian Diseases, College of Veterinary Medicine, Sichuan Agricultural University, Wenjiang, Chengdu, Sichuan 611130 P.R. China; 2 Key Laboratory of Animal Disease and Human Health of Sichuan Province, Wenjiang, Chengdu, Sichuan 611130 P.R. China; 3 Institute of Preventive Veterinary Medicine, Sichuan Agricultural University, Wenjiang, Chengdu, Sichuan 611130 P.R. China; 4 Center for Infectious Diseases and Vaccinology, The Biodesign Institute, Arizona State University, Tempe, AZ 85287-5401 USA

## Abstract

Non-typhoidal *Salmonella* are associated with gastrointestinal disease worldwide and invasive disease in Africa. We constructed novel bivalent vaccines through the recombinant expression of heterologous O-antigens from *Salmonella* Choleraesuis in *Salmonella* Typhimurium. A recombinant Asd^+^ plasmid pCZ1 with the cloned *Salmonella* Choleraesuis O-antigen gene cluster was introduced into three constructed *Salmonella* Typhimurium Δ*asd* mutants: SLT11 (Δ*rfbP*), SLT12 (Δ*rmlB*-*rfbP*) and SLT16 (Δ*rfbP* ∆*pagL*::TT *araC*P_BAD_ *rfbP*). Immunoblotting demonstrated that SLT11 (pCZ1) and SLT12 (pCZ1) efficiently expressed the heterologous O-antigen. In the presence of arabinose, SLT16 (pCZ1) expressed both the homologous and heterologous O-antigens, whereas in the absence of arabinose, SLT16 (pCZ1) mainly expressed the heterologous O-antigen. We deleted the *crp*/*cya* genes in SLT12 (pCZ1) and SLT16 (pCZ1) for attenuation purposes, generating the recombinant vaccine strains SLT17 (pCZ1) and SLT18 (pCZ1). Immunization with either SLT17 (pCZ1) or SLT18 (pCZ1) induced specific IgG against the heterologous O-antigen, which mediated significant killing of *Salmonella* Choleraesuis and provided full protection against a lethal homologous challenge in mice. Furthermore, SLT17 (pCZ1) or SLT18 (pCZ1) immunization resulted in 83% or 50% heterologous protection against *Salmonella* Choleraesuis challenge, respectively. Our study demonstrates that heterologous O-antigen expression is a promising strategy for the development of multivalent *Salmonella* vaccines.

## Introduction

Non-typhoidal *Salmonella* (NTS), a group of Gram-negative and facultative intracellular bacteria, are important zoonotic pathogens that cause foodborne gastrointestinal disease in humans and animals and pose a public health burden worldwide^1, 2^. It is estimated that 93.8 million cases of gastroenteritis occur due to NTS worldwide, leading to 155,000 diarrheal deaths each year^3^. NTS can also cause invasive diseases, such as bacteremia, septicemia and meningitis, with high morbidity and mortality in high risk individuals in industrialized and developing countries and in young children in sub-Saharan Africa^4, 5^. However, there is no vaccine currently available against NTS serovars in humans, although some O-antigen-based conjugate vaccines, live attenuated vaccines and outer membrane protein (OMP)-based subunit vaccines are in development.

Exposure to animal and animal food products is one of the major risk factors resulting in human salmonellosis^6–8^. NTS comprise more than 2500 different serovars; however, relatively few restricted serovars are associated with bacterial transmission between animals and humans. These serovars include *Salmonella enterica* serovar Typhimurium (*Salmonella* Typhimurium), *Salmonella* Enteritidis and Group C *Salmonella*, such as *Salmonella* Choleraesuis and *Salmonella* Newport^2, 9, 10^. Hence, a significant burden is placed on both the animal industry and the healthcare system due to these bacteria. Furthermore, with the increasing frequencies of multi-drug resistant *Salmonella* strains, antibiotic treatment in human patients is becoming increasingly difficult^11, 12^. Thus, there is growing recognition that an ideal multivalent vaccine with broad coverage of epidemic serovars is needed to control NTS infections in both animals and humans.

The lipopolysaccharide (LPS) O-antigen, which is expressed on the outer surface of the bacterium and consists of oligosaccharide repeats, is the basis for the typing system of *Salmonella* serovars together with the flagellar antigen and determines the serogroup^13, 14^. The O-antigen is highly immunogenic and has become an important target of protective immunity^15^. Passive transfer of monoclonal IgG specific to the O-antigen of *Salmonella* Typhimurium conferred protection against virulent *Salmonella* Typhimurium challenge^16, 17^. A protective monoclonal IgA (named Sal4) against the O-antigen of *Salmonella* Typhimurium could impair type 3 secretion and outer membrane integrity, rendering the bacteria avirulent^18^. The key role of the O-antigen in protective immunity is to promote the development of O-antigen-based conjugate vaccines, such as O:9-flagellin^19, 20^, O:4,12-TT^21^, O:4,5/O:9-CRM197^22^ and Os-po^23^. All these conjugate vaccines have provided protection against virulent challenge in preclinical studies; nevertheless, they protect against only serovars with the same O-antigen specificity. Although cross-protection may be achieved by mixing these conjugate vaccines, the increasing cost of this approach is a disadvantage for application of the glycoconjugate technology^24^. Additionally, some attenuated *Salmonella* vaccines have exhibited cross-protective efficacy against different serovars^25, 26^; however, the cross-efficacy is limited, and only partial protection is achieved after challenge with virulent strains of heterologous serovars. Thus, there is an urgent need for novel approaches for developing a multivalent vaccine.

Live attenuated *Salmonella* Typhimurium has been extensively used as a vaccine vehicle to deliver heterologous antigens to the immune system and stimulate a protective immune response against a variety of targeted pathogens at a low cost^27^. A series of technologies, including the balanced-lethal vector-host systems and the regulated delayed *in vivo* attenuation, have been developed in *Salmonella* to improve the potency of recombinant vaccines^28^. We hypothesized that immunization with a live attenuated *Salmonella* Typhimurium expressing heterologous O-antigens of other serovars in the outer membrane would provide both homologous and heterologous protection. The O-antigen gene cluster of *Salmonella* serovars responsible for O-antigen biosynthesis has been identified^13^. Here, we introduced the O-antigen gene cluster of *Salmonella* Choleraesuis, which resides on the Asd^+^ plasmid pCZ1, into three *Salmonella* Typhimurium Δ*asd* strains: SLT11 (Δ*asd* Δ*rfbP*), SLT12 (Δ*asd* Δ*rmlB*-*rfbP*) and the arabinose-regulated strain SLT16 (Δ*asd* Δ*rfbP* ∆*pagL*::TT *araC*P_BAD_ *rfbP*), and we then verified the expression of the homologous and heterologous O-antigens in the three recombinant strains. Because *Salmonella* O-antigen also plays a vital role in bacterial survival and virulence^29–31^, we first determined the effects of heterologous O-antigen expression on several phenotypes, including swimming, sensitivities to polymyxin B and sodium deoxycholate (DOC) and colonization. We then introduced *crp* and *cya* gene mutations into the SLT12 (pCZ1) and SLT16 (pCZ1) strains for attenuation, and finally, we measured the immunogenicity and protective efficacy of the recombinant attenuated vaccine strains of *Salmonella* Typhimurium in BALB/c mice.

## Results

### Construction and characterization of recombinant *Salmonella* Typhimurium strains

The *asd*-based balanced-lethal vector-host system^32^ was used to stably express heterologous O-antigen in *Salmonella* Typhimurium. The Asd^+^ plasmid pCZ1 was constructed by overlapping DNA elements, including the pSC101 origin, *asd* gene cassette, TIT2 terminator, Ptrc promoter, kanamycin-resistance cassette from the plasmid pQK664^33^ and the whole O-antigen gene cluster of *Salmonella* Choleraesuis. Moreover, to block the synthesis of the *Salmonella* Typhimurium O-antigen, the *rfbP* gene (responsible for the addition of the Gal-1-phosphate residue to UndP to initiate O-unit synthesis) or the whole O-antigen gene cluster (*rmlB*-*rfbP*) was deleted from the *Salmonella* Typhimurium strain SLT10 (S100 Δ*asd*), generating SLT11 (SLT10 Δ*rfbP*) or SLT12 (SLT10 Δ*rmlB*-*rfbP*), respectively. In addition, we used arabinose-dependent regulated delayed attenuation systems^34, 35^ to construct strain SLT16 (SLT10 Δ*rfbP* ∆*pagL*::TT *araC*P_BAD_ *rfbP*) by replacing the *rfbP* promoter with the *araC*P_BAD_ promoter to ensure that the expression of homologous *Salmonella* Typhimurium O-antigen was activated by exogenous arabinose provided during *in vitro* growth^34^. Then, pCZ1 was introduced into SLT11, SLT12 and SLT16 to complete the construction of the three recombinant *Salmonella* strains SLT11 (pCZ1), SLT12 (pCZ1) and SLT16 (pCZ1). The control strains SLT11 (pQK664), SLT12 (pQK664) and SLT16 (pQK664) were also constructed.

**Figure 1:**
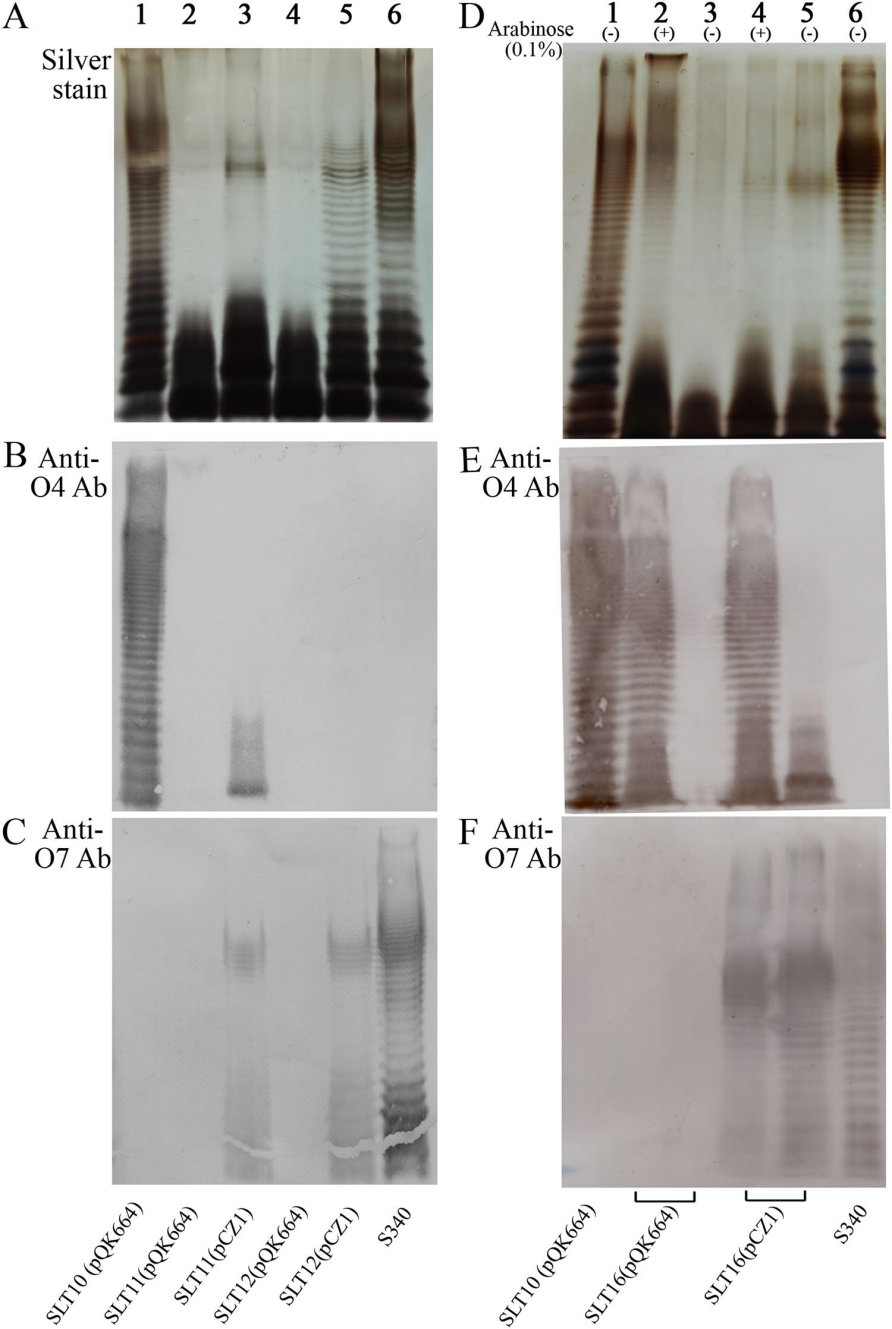
LPS analysis by silver-stain and Western immunoblotting. (**A**,**B** and **C**). LPS extracted from *Salmonella* Typhimurium SLT10 (pQK664) (lane 1), SLT11 (pQK664) (lane 2), SLT11 (pCZ1) (lane 3), SLT12 (pQK664) (lane 4), SLT12 (pCZ1) (lane 5) and *Salmonella* Choleraesuis S340 (lane 6) was subjected to SDS-PAGE followed by silver staining (**A**) and immunoblotting analysis using O:4-specific antisera (**B**) and O:7-specific antisera (**C**). (**D**,**E** and **F**) LPS extracted from SLT10 (pQK664) (lane 1), SLT16 (pQK664) grown with arabinose (lane 2) or without arabinose (lane 3), SLT16 (pCZ1) grown with arabinose (lane 4) or without arabinose (lane 5) and S340 (lane 6) was subjected to SDS-PAGE followed by silver staining (**D**) and immunoblotting using O:4-specific antisera (**E**) and O:7-specific antisera (**F**). Each lane corresponds to LPS from 10^8^ bacteria.

LPS analysis by silver staining and Western immunoblotting was employed for the parent and recombinant strains, which were cultured and adjusted to the same optical density before analysis. Silver staining showed that the *Salmonella* Typhimurium parent strain SLT10 and the *Salmonella* Choleraesuis wild-type strain S340 produced complete LPS ladders (Fig. 1A, lanes 1 and 6). As expected, SLT11 (pCZ1) and SLT12 (pCZ1) displayed smooth LPS phenotypes (Fig. 1A, lanes 3 and 5), whereas SLT11 (pQK664) and SLT12 (pQK664) displayed rough LPS phenotypes (Fig. 1A, lanes 2 and 4). Western immunoblotting with O:4- and O:7-specific antisera demonstrated that SLT12 (pCZ1) expressed the heterologous O-antigen but not the homologous O-antigen (Fig. 1B and C, lane 5); however, unexpectedly, SLT11 (pCZ1) expressed not only the heterologous O-antigen but also short-length homologous O-antigen (Fig. 1B and C, lane 3). SLT16 (pCZ1) expressed O-antigens in an arabinose-dependent manner (Fig. 1D,E and F). In the presence of arabinose, SLT16 (pCZ1) expressed both the complete homologous and heterologous O-antigens (Fig. 1E and F, lane 4), whereas in the absence of arabinose, SLT16 (pCZ1) expressed the heterologous O-antigen and short-length homologous O-antigen (Fig. 1E and F, lane 5). Thus, SLT16 (pCZ1) displayed a chimeric O-antigen profile. In contrast, the control strain SLT16 (pQK664) expressed homologous O-antigen but not heterologous O-antigen when grown with arabinose (Fig. 1E and F, lane 2) and did not express either the homologous or heterologous O-antigen when grown without arabinose (Fig. 1E and F, lane 3). Because SLT12 (pCZ1) expressed more heterologous antigen than SLT11 (pCZ1) (Fig. 1A, lane 3 vs lane 5), SLT12 (pCZ1) and SLT16 (pCZ1) were used for further biological analyses.

### Effects of heterologous O-antigen expression on bacterial phenotypes

**Figure 2:**
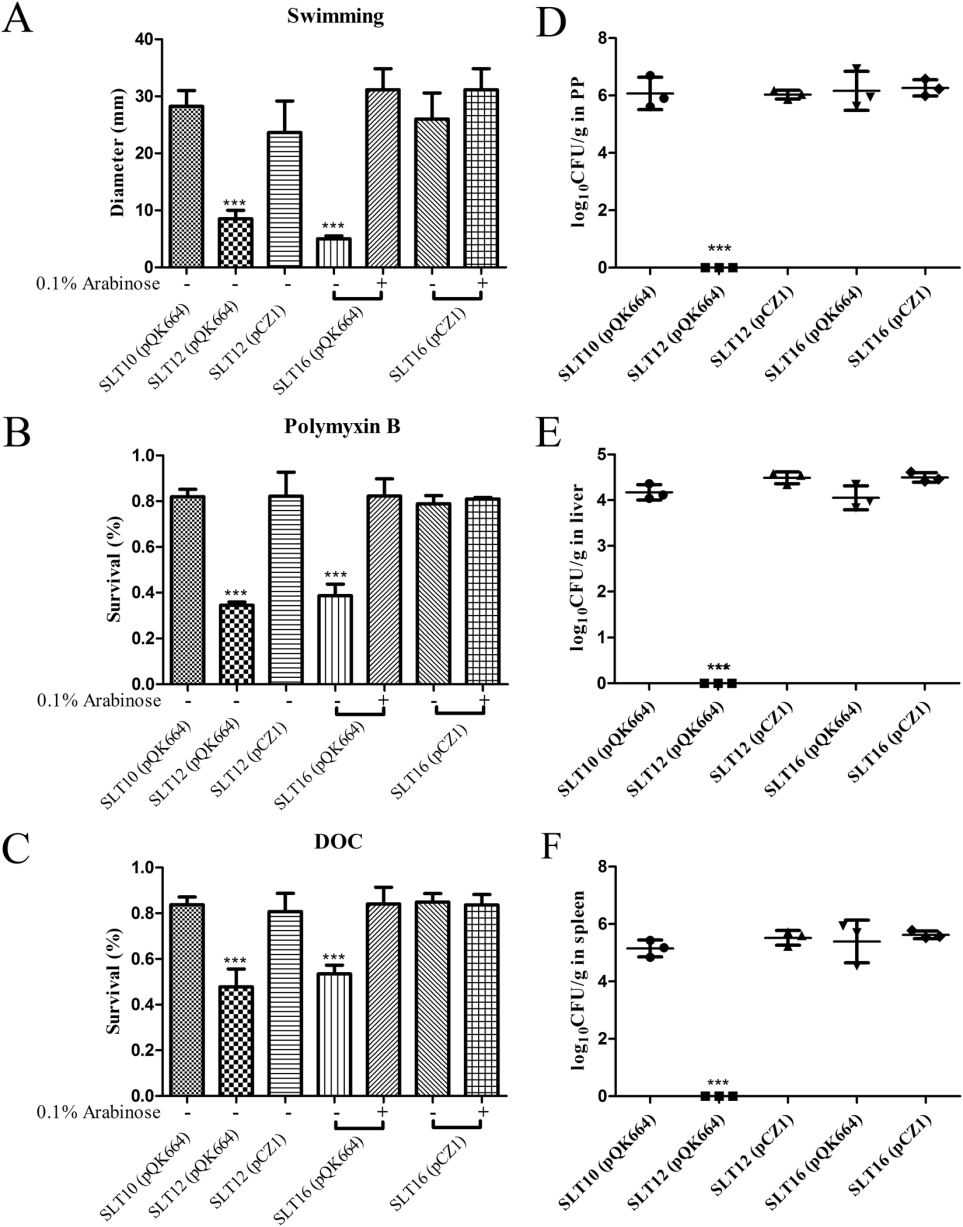
Effects of heterologous O-antigen expression on *Salmonella* Typhimurium biological activities. (**A**) Swimming assay. SLT10 (pQK664), SLT12 (pQK664), SLT12 (pCZ1) and the two arabinose-regulated strains SLT16 (pQK664) and SLT16 (pCZ1) were grown in LB broth with or without 0.1% arabinose. Then, 6 μl of the bacterial suspension (approximately 1 × 10^6^ CFU) was spotted onto the middle of LB plates containing 0.3% agar with or without arabinose, and the diameter of the colonies was measured 6 h after incubation. (**B** and **C**) Resistance to polymyxin and DOC. The same *Salmonella* Typhimurium strains described in A were cultured in LB broth with or without 0.1% arabinose. One-hundred microliters of the cell suspension (approximately 1 × 10^6^–5 × 10^6^ CFU) was inoculated with or without 0.12 μg/ml polymyxin B (**B**) or 4 mg/ml DOC (**C**) for 1 h at 37 °C. The bacteria were diluted and plated onto LB plates, and the survival rate was calculated the following day. (**D**,**E** and **F**) Colonization. Groups of BALB/c mice (n = 4/group) were orally inoculated with 1 × 10^9^ CFU of each indicated strain. Viable bacteria were recovered from PP (**D**), liver (**E**) and spleen (**F**) 6 days after infection. The bacterial number in each tissue was calculated as log_10_ CFU/g. The asterisk above the error bar indicates significance compared to the SLT10 (pQK664) group. ***p < 0.001.

Because the O-antigen plays an important role in *Salmonella* Typhimurium colonization and survival processes^31^, we evaluated whether the altered O-antigen profiles could affect bacterial phenotypes, including swimming, resistance to polymyxin B and DOC and colonization *in vivo*. As shown in Fig. 2A, the rough strains SLT12 (pQK664) and SLT16 (pQK664) (grown without arabinose) showed a defective swimming phenotype, while SLT12 (pCZ1), SLT16 (pQK664) (grown with arabinose) and SLT16 (pCZ1) (grown with or without arabinose) with the smooth LPS phenotype showed a similar motility capacity as the parent strain SLT10 (pQK664). A similar result was observed in experiments examining resistance to polymyxin B and DOC. The strains SLT12 (pCZ1), SLT16 (pCZ1) and SLT16 (pQK664) (grown with arabinose) exhibited comparable resistance to polymyxin B and DOC as the parent strain (Fig. 2B and C), while SLT12 (pQK664) and SLT16 (pQK664) (grown without arabinose) exhibited decreased resistance to polymyxin B and DOC (Fig. 2B and C). In addition, SLT12 (pCZ1), SLT16 (pCZ1) and SLT16 (pQK664) displayed colonization levels in Peyer’s Patches (PP), liver and spleen of BALB/c mice that were comparable to the parent strain (Fig. 2D,E and F). Unexpectedly, no bacteria were detected in the three tissues after infection with the rough strain SLT12 (pQK664) (Fig. 2D,E and F); thus, SLT12 (pQK664) colonization was significantly decreased compared to that of the parent strain. Therefore, our results demonstrated that heterologous O-antigen expression does not affect bacterial swimming, resistance to polymyxin B and DOC or colonization.

### Construction of the recombinant vaccine strains and virulence evaluation

**Figure 3:**
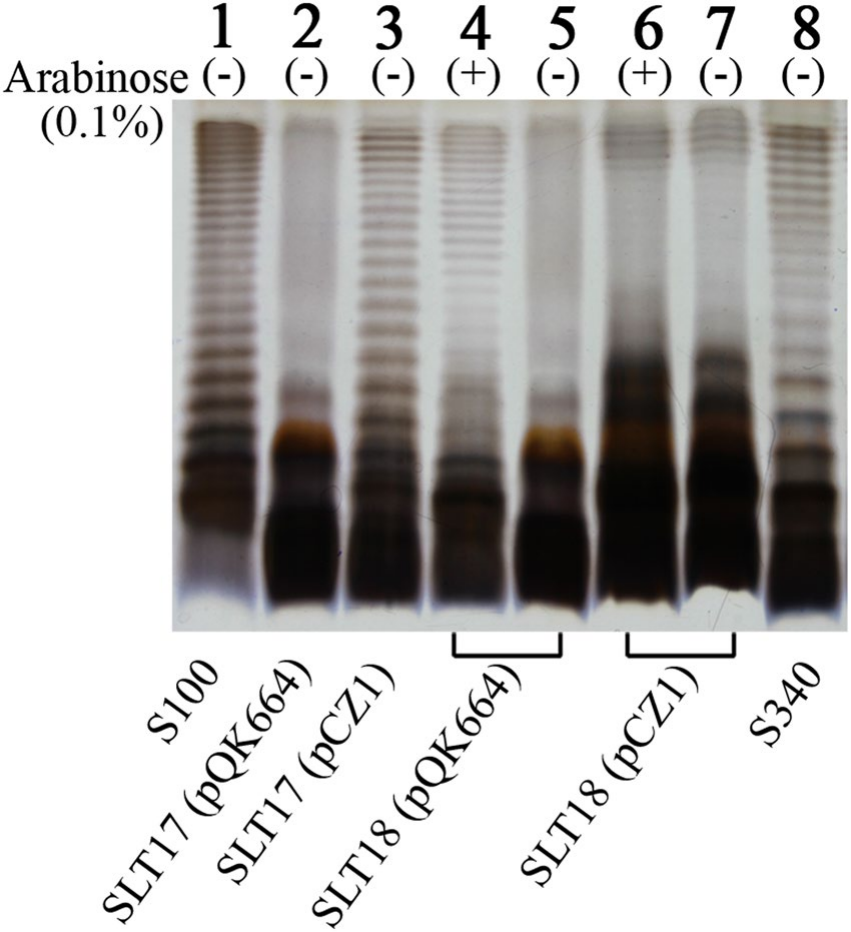
LPS phenotypes of the vaccine strains. LPS samples extracted from *Salmonella* Typhimurium wild-type S100 (lane 1), SLT17 (pQK664) (lane 2), SLT17 (pCZ1) (lane 3) and SLT18 (pQK664) grown with arabinose (lane 4) or without arabinose (lane 5), SLT18 (pCZ1) grown with arabinose (lane 6) or without arabinose (lane 7) and *Salmonella* Choleraesuis S340 (lane 8) were visualized by silver staining following PAGE. Each lane corresponds to LPS from 10^8^ bacteria.

The *crp* and *cya* genes were deleted from SLT12 (pQK664), SLT12 (pCZ1), SLT16 (pQK664) and SLT16 (pCZ1) to attenuate virulence, generating the two recombinant vaccine strains SLT17 (pCZ1) and SLT18 (pCZ1) and the two control vaccine strains SLT17 (pQK664) and SLT18 (pQK664). Silver staining and Western immunoblotting showed that SLT17 (pCZ1) or SLT18 (pCZ1) displayed a similar LPS phenotype as SLT12 (pCZ1) or SLT16 (pCZ1) (Figs 1 and 3 and Supplementary Fig. S1); the control vaccine strains SLT17 (pQK664) and SLT18 (pQK664) (grown without arabinose) showed rough LPS phenotypes (Fig. 3 and Supplementary Fig. S1, lanes 2 and 5). The silver staining also showed that SLT17 (pCZ1) expressed more heterologous O-antigens than SLT18 (pCZ1) (grown without arabinose) (Fig. 3, lane 3 vs lane 7). Furthermore, the 50% lethal dose (LD_50_) and colonization for each of the four vaccine strains were determined in BALB/c mice. All vaccine strains were avirulent, and their LD_50_ values were more than 2 × 10^9^ CFU, which was > 10^4^-fold higher than that of the wild-type strain S100 (LD_50_ of 5.4 × 10^5^ CFU) (Supplementary Table S1). The colonization levels of all of the vaccine strains in PP, liver and spleen were significantly decreased compared with those of the wild-type S100 strain (Supplementary Fig. S2). The vaccine strains SLT17 (pCZ1), SLT18 (pQK664) and SLT18 (pCZ1) retained similar capacities to efficiently colonize mouse tissues. In contrast, no bacteria were detected in the three tissues after infection with the control strain SLT17 (pQK664) (Supplementary Fig. S2).

### Immunogenicity and protective efficacy of the recombinant attenuated vaccine strains

**Figure 4:**
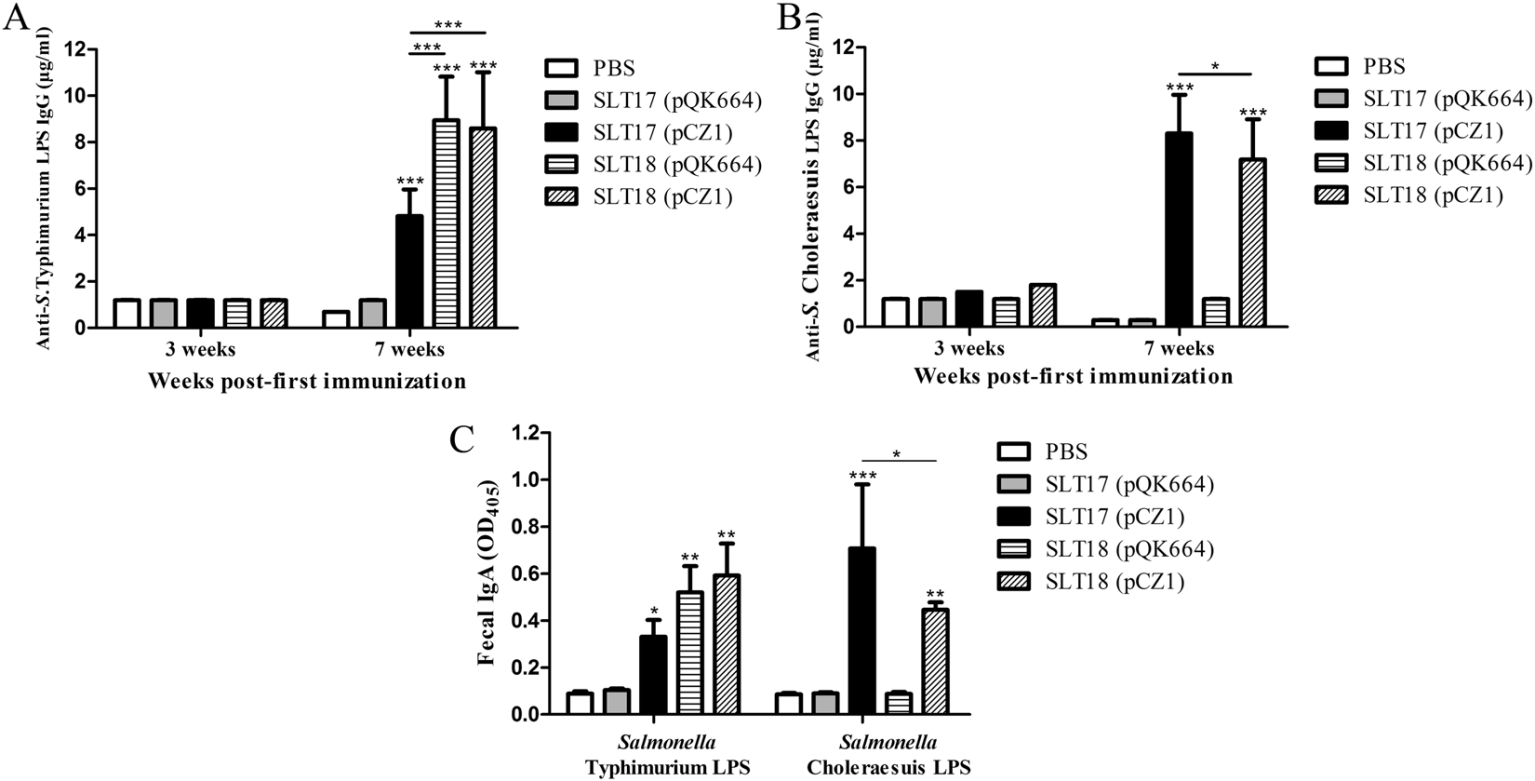
Antibody responses induced by the recombinant attenuated vaccines. (**A** and **B**) Serum IgG responses. Groups of BALB/c mice were immunized with 1 × 10^9^ CFU of SLT17 (pQK664), SLT17 (pCZ1), SLT18 (pQK664), SLT18 (pCZ1) or PBS twice at an interval of 4 weeks. Serum samples were collected from 6 mice from each group 3 weeks and 7 weeks after the first immunization. The serum IgG specific to *Salmonella* Typhimurium LPS (**A**) and *Salmonella* Choleraesuis LPS (**B**) was measured by quantitative ELISA in each group (**C**). Fecal IgA responses. The fecal samples were collected from 6 mice of each group 3 weeks after the second immunization. Fecal IgAs against *Salmonella* Typhimurium LPS and *Salmonella* Choleraesuis LPS were detected by indirect ELISA. The asterisk above the error bar indicates significance compared with the PBS control group. The asterisk above the line indicates significance between the two indicated groups. *p < 0.05. **p < 0.01. ***p < 0.001.

Seven-week-old BALB/c mice were orally immunized with PBS or approximately 10^9^ CFU of each vaccine strain twice at an interval of four weeks. The mice were then challenged orally with the virulent strains *Salmonella* Typhimurium S100^36^ and *Salmonella* Choleraesuis S340^36^ at a dose of at least 100 × LD_50_ one month after the second immunization. Serum IgG and fecal IgA antibodies against either the homologous *Salmonella* Typhimurium or heterologous *Salmonella* Choleraesuis LPS were detected by enzyme-linked immunosorbent assay (ELISA) after immunization. No specific serum IgG antibody against either the homologous or heterologous LPS was induced after the first immunization in any of the immunized groups (Fig. 4A and B). All vaccine strains except SLT17 (pQK664) stimulated significantly higher levels of serum IgG and fecal IgA against *Salmonella* Typhimurium LPS than the PBS control after the second immunization (Fig. 4A and C), and the IgG levels induced by the vaccine strains SLT18 (pQK664) and SLT18 (pCZ1) were significantly higher than that of the vaccine strain SLT17 (pCZ1) (Fig. 4A). Furthermore, only the recombinant vaccine strains SLT17 (pCZ1) and SLT18 (pCZ1) induced a specific serum IgG or fecal IgA response against *Salmonella* Choleraesuis LPS after the second immunization, and the IgG or IgA level induced by SLT17 (pCZ1) was significantly higher than that of SLT18 (pCZ1) (Fig. 4B and C).

**Figure 5:**
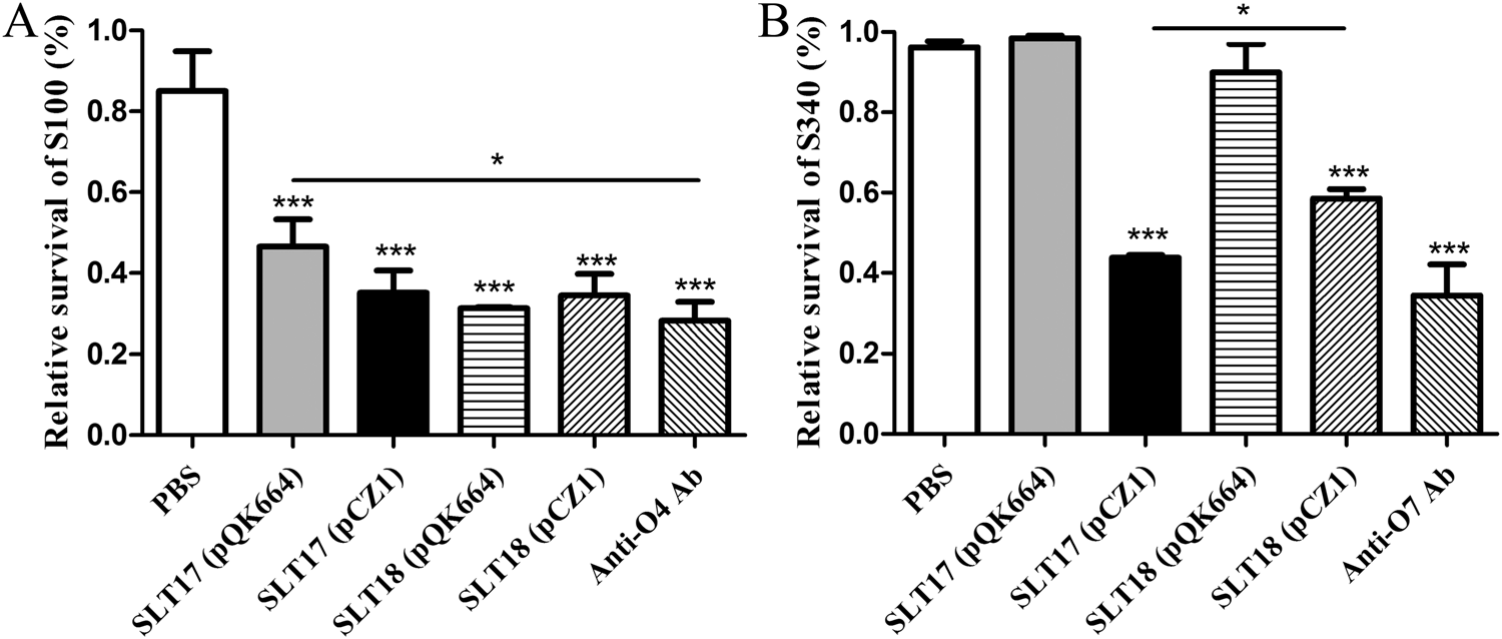
Serum bactericidal assay. *Salmonella* Typhimurium S100 (**A**) or *Salmonella* Choleraesuis S340 (**B**) was incubated with the serum collected from each immunized group at 3 weeks post-second immunization. Active guinea pig complement was added to or omitted from the mixture. The relative survival was calculated at 1.5 h post-incubation. The asterisk above the error bar indicates significance compared to the PBS control group. The asterisk above the line indicates significance between the two indicated groups. *p < 0.05. ***p < 0.001.

To determine whether each vaccine strain elicited bactericidal antibodies, we used the serum from each group at week 3 post-second immunization and measured the relative bactericidal activities against the homologous *Salmonella* Typhimurium strain S100 and the heterologous *Salmonella* Choleraesuis strain S340. The bacteria were incubated with 25% heat-inactivated serum plus active guinea pig complement or no complement for 1.5 h, and the relative survival was then calculated as the percent CFU in each serum with active complement compared to the CFU of the same serum with no complement. *Salmonella* O:4-specific and O:7-specific antisera were included as positive controls. As shown in Fig. 5A, the antibodies elicited by either the recombinant strains SLT17 (pCZ1) and SLT18 (pCZ1) or the control strains SLT17 (pQK664) and SLT18 (pQK664) were able to kill the homologous S100 strain at a similar level, which was significantly better than the antibodies from the PBS group. The ability of positive O:4-specific antisera to mediate killing was comparable to that of the antisera induced by the SLT17 (pCZ1), SLT18 (pQK664) and SLT18 (pCZ1) groups but better than that of the serum induced by SLT17 (pQK664) (Fig. 5A). Furthermore, the antibody from the SLT17 (pCZ1) and SLT18 (pCZ1) groups resulted in significant killing against the heterologous S340 strain, which was much better than that of the PBS group and comparable to that of the positive O:7-specific antisera (Fig. 5B). In contrast, no significant killing against the heterologous S340 strain was observed in the control SLT17 (pQK664) or SLT18 (pQK664) groups. Notably, the relative survival of S340 in the SLT17 (pCZ1) group was significantly lower than that of the SLT18 (pCZ1) group (Fig. 5B), indicating that SLT17 (pCZ1) immunization induces enhanced serum bactericidal effects.

**Figure 6:**
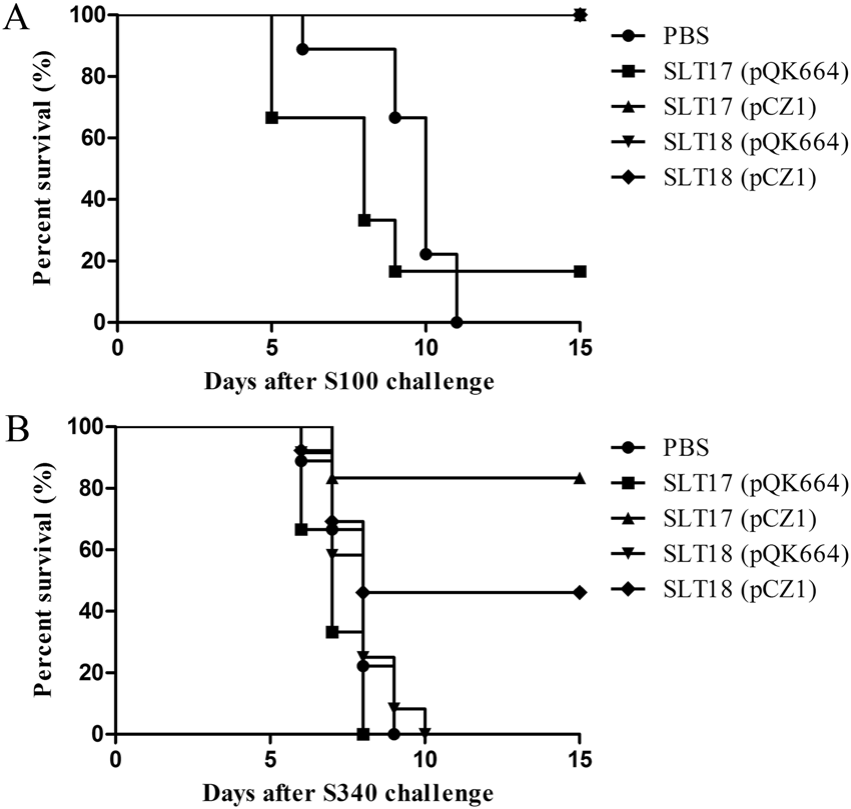
Survival rates after a lethal oral challenge with *Salmonella* Typhimurium S100 (**A**) or *Salmonella* Choleraesuis S340 (**B**) are shown. The immunized mice (n = 12/group) were challenged with at least 100 × LD_50_ of virulent S100 or virulent S340. Mortality was recorded daily for 15 days.

All 12 mice in the SLT18 (pQK664), SLT17 (pCZ1) and SLT18 (pCZ1) immunized groups survived the *Salmonella* Typhimurium S100 challenge, suggesting 100% protection. In contrast, only 2 of 12 mice in the SLT17 (pQK664) group survived the challenge, and all 12 mice of the PBS group succumbed to the challenge (Fig. 6A). In the presence of the *Salmonella* Choleraesuis S340 challenge, the SLT17 (pCZ1) immunization resulted in 83% survival, which was better than the 50% survival observed for the SLT18 (pCZ1) group (Fig. 6B, log-rank test, p < 0.01). All of the mice in the control SLT17 (pQK664), SLT18 (pQK664) and PBS groups succumbed to the challenge (Fig. 6B). Thus, the recombinant vaccine strains SLT17 (pCZ1) and SLT18 (pCZ1) provided full protection against lethal homologous challenge and 83% and 50% heterologous protection against *Salmonella* Choleraesuis lethal challenge, respectively.

## Discussion

NTS serovars Typhimurium, Enteritidis and Group C *Salmonella* are important causes of foodborne salmonellosis worldwide^3^. There is urgent need for an effective vaccine with broad serovar coverage for the prevention of salmonellosis in humans and animals. Although some types of vaccines, such as live attenuated vaccines and O-antigen-based conjugate vaccines, are in development^37^, there is no or only partial cross-protection achieved by these vaccines. Here, we developed two bivalent vaccines, SLT17 (pCZ1) and SLT18 (pCZ1), by recombinant expression of *Salmonella* Choleraesuis O-antigen (Group C1) in attenuated *Salmonella* Typhimurium strains. The reason for the selection of *Salmonella* Typhimurium as the vaccine vector rather than other serovars is that *Salmonella* Typhimurium is the most well-studied NTS serovar, and a set of recombinant vaccine technologies has been developed for this serovar^28^. Here, we used the Asd-based balanced-lethal vector-host system^32^ to maintain the expression of the heterologous O-antigen and an arabinose-dependent regulated attenuation system^34^ to achieve expression of the homologous O-antigen dependent on exogenously supplied arabinose during *in vitro* growth.

O-antigens are characterized as immunodominant and protective antigens in *Salmonella* as well as in several other infectious bacteria. Recombinant attenuated *Salmonella* vaccines expressing *Shigella sonnei* O-antigen or *Burkholderia mallei* O-antigen have been constructed and exhibit protection against lethal challenge^38, 39^. Surprisingly, a similar strategy has not been used for the development of bivalent or multivalent *Salmonella* vaccines. A potential advantage of O-antigen-based *Salmonella* vaccines is that they should offer cross-protection against other serovars within the same serogroup because all serovars in one serogroup express the same dominant O-antigen epitopes^24^ (group C1 serovars all express O:6,7 antigen^13^). Antigen level is an important factor influencing the magnitude of the immune response. To maximize the expression of the heterologous *Salmonella* Choleraesuis O-antigen, synthesis of the *Salmonella* Typhimurium O-antigen (Group B1) was blocked by deletion of either the *rfbP* gene or the whole O-antigen gene cluster. The pCZ1 plasmid containing the whole O-antigen gene cluster of *Salmonella* Choleraesuis was then introduced into the rough mutant strains. This strategy for gene deletion and introduction was based on the finding that the Group C1 O-antigen and its gene cluster are quite distinct from the Group B1 O-antigen^13^. As expected, the two recombinant strains, SLT11 (pCZ1), with deletion of the *rfbP* gene, and SLT12 (pCZ1), with deletion of the whole *Salmonella* Typhimurium O-antigen gene cluster, efficiently expressed the heterologous O-antigen, whereas unexpectedly, SLT11 (pCZ1) also expressed the homologous O-antigen, as indicated by the immunoblotting results (Fig. 1B and C, lanes 3 and 5). Because the genes for initiating O-unit synthesis in Groups C1 and B1 were different (*wecA* for C1 O-antigen and *rfbP* for B1 O-antigen)^13^ and the homologous O-antigen expression was not observed in SLT11 (pQK664) (Fig. 1B and C, lane 2), it was impossible to express the complete *Salmonella* Typhimurium O-antigen structure in SLT11 (pCZ1). According to the O-antigen structures and the required glycosyltransferase genes between Groups B1 and C1^13^, we speculate that a branching sugar (abequose) was synthesized and attached to D-mannose in the backbone of heterologous O-antigen in SLT11 (pCZ1) by the *abe* and *wbaV* genes, which reside in the *Salmonella* Typhimurium O-antigen gene cluster, conferring immunodominant epitope 4 specificity^15^, which can be recognized by the *Salmonella* O:4 specific antisera that was employed. In contrast, the band specifying the *Salmonella* Typhimurium O-antigen was not observed in SLT12 (pCZ1) (Fig. 1B, lane 5) because the entire *Salmonella* Typhimurium O-antigen gene cluster was deleted. Therefore, it was possible that the SLT11 (pCZ1) expressed the O:4 epitope rather than the complete homologous O-antigen. Given that the antibodies specific to the homologous O-antigen play important roles in immune protection against *Salmonella* Typhimurium^31^, we constructed another recombinant strain, SLT16 (pCZ1), in which the heterologous O-antigen and complete homologous O-antigen were expressed when arabinose was available; the heterologous O-antigen and short-length homologous (probably the O:4 epitope) were expressed when grown without arabinose, similar to that displayed by the SLT11 (pCZ1) (Fig. 1D,E and F). Because arabinose is not present in tissues upon the invasion of *Salmonella* into the gut-associated lymphoid tissue^40^, the expression of complete homologous O-antigen was activated before the bacteria invaded these cells and ceased after invasion due to cell division.

The O-antigen is an important virulence factor, which is involved in bacterial motility, colonization and survival in the host^29, 31^. Loss of the entire O-antigen attenuates swimming motility and intestinal colonization and renders bacteria highly susceptible to antimicrobial agents. In our constructed recombinant strains SLT12 (pCZ1) and SLT16 (pCZ1), the wild-type *Salmonella* Typhimurium O-antigen was replaced by the heterologous O-antigen or the chimeric O-antigen. We determined whether the altered O-antigen structures influenced swimming motility, resistance to polymyxin B and DOC and colonization of *Salmonella* Typhimurium. The results showed that none of the examined phenotypes were affected by heterologous O-antigen expression (Fig. 2), indicating that O-antigen expression itself, rather than O-antigen variety, was vital for these *Salmonella* phenotypes. Additionally, the rough strains SLT12 (pQK664) and SLT16 (pQK664) (grown without arabinose) showed decreased swimming and increased sensitivity to polymyxin B and DOC, which was consistent with previous studies^29^. Surprisingly, no detectable bacteria were recovered from the PP, liver or spleen tissues 6 days after oral inoculation with STL12 (pQK664) (Fig. 2D,E and F). The attenuated colonization is consistent with previous results showing that deletion of genes responsible for O-antigen synthesis, such as *rfaH* and *rfbP*, results in rough LPS and decreased but not undetectable colonization^31^. This result suggested that deletion of the whole O-antigen cluster further attenuates colonization.

Next, the *crp* and *cya* genes, which have been identified as the mutation targets of live vaccines^41, 42^, were deleted from the recombinant and control strains for attenuation and to evaluate the immunogenicity and protective efficacy. The derived vaccine strains showed similar LPS phenotypes as their parent strains (Figs 3 and S1), suggesting that deletions of *crp* and *cya* do not affect LPS structure, in agreement with previous reports^43^. Both the recombinant vaccine strains SLT17 (pCZ1) and SLT18 (pCZ1) induced significantly higher serum IgG and fecal IgA responses against the homologous and heterologous LPS than the PBS control at 3 weeks post-second immunization (Fig. 4). The bactericidal effects exerted by sera induced by the two recombinant vaccine strains were comparable to those of the homologous S100 strain with the O:4-specific antisera (Fig. 5A) and the heterologous S340 strain with the O:7-specific antisera (Fig. 5B). The control strain SLT17 (pQK664) did not produce antibodies immunoreactive to either the homologous or heterologous LPS (Fig. 4), possibly due to a combination of the rough LPS phenotype (Supplementary Fig. S1) and undetectable colonization in mouse tissues (Supplementary Fig. S2); however, the serum from the SLT17 (pQK664)-immunized group mediated significantly increased killing of the homologous S100 strain compared with the PBS group. Combined with the finding that immunization with SLT17 (pQK664) induced low-level homologous protection (Fig. 6A), this result suggested that SLT17 (pQK664) was still immunogenic and induced a specific antibody response against other *Salmonella* Typhimurium antigens with high immunogenicity rather than the rough LPS. Furthermore, the other control strain, SLT18 (pQK664), which expressed the homologous O-antigen only when arabinose was present during *in vitro* growth, produced significant serum IgG and fecal IgA specific to the homologous LPS but not to the heterologous LPS (Fig. 4). In addition, serum produced from SLT18 (pQK664) exhibited killing against the homologous S100 strain but not the heterologous S340 strain (Fig. 5A and B), suggesting that there was less cross-immunoreactivity between serum antibodies specific to *Salmonella* Typhimurium and *Salmonella* Choleraesuis. However, SLT17 (pCZ1), which expressed only the heterologous O-antigen, elicited detectable anti-homologous LPS IgG and IgA (Fig. 4A and C), similar to the phenomenon that was observed in immunization experiments based on the *Salmonella* Typhimurium Δ*rfbP* mutant with a rough LPS phenotype^31^. We speculate that the antibodies detected were in response to the core region or lipid A of *Salmonella* Typhimurium LPS, which were contained in the ELISA coating antigens.

The potent antibody responses specific to the heterologous O-antigen and its functional bactericidal effects against *Salmonella* Choleraesuis induced by the two recombinant vaccines SLT17 (pCZ1) and SLT18 (pCZ1) demonstrated the high immunogenicity of the delivered heterologous O-antigen. To determine whether the induced antibody responses conferred heterologous protection, the protective efficacy was further investigated. The vaccine strains SLT17 (pCZ1), SLT18 (pQK664) and SLT18 (pCZ1) provided full homologous protection against lethal challenge with *Salmonella* Typhimurium (Fig. 6A), which was in line with the previous vaccine studies based on the *crp/cya* mutant and validated the immunization and challenge conditions of our study. The full homologous protection conferred by the SLT17 (pCZ1), which did not express the homologous O-antigen, was in line with the finding observed in previous studies, which showed that attenuated *Salmonella* Typhimurium Δ*rfaH* or Δ*rmlB* mutant with rough LPS phenotypes also exhibited protection to homologous lethal challenge^30, 36^. Combined with the finding that the serum induced by SLT17 (pCZ1) exhibited significant bactericidal effects to the homologous S100 strain, the result suggested that the O-antigen-mediated antibody response was not indispensable for preventing *Salmonella* infections, and immune responses specific for other *Salmonella* antigens, such as OMPs, support the efficacy of these live *Salmonella* vaccines with rough LPS phenotypes^36^. In contrast, the control SLT17 (pQK664) strain mediated inefficient homologous protection (Fig. 6A), which is likely due to the undetectable colonization (Supplementary Fig. S2) and the resulting poor immunogenicity. In comparison with the full homologous protection provided by SLT18 (pQK664), no heterologous protection was observed in the SLT18 (pQK664) control groups (Fig. 6B), indicating little cross-protection between *Salmonella* Typhimurium and *Salmonella* Choleraesuis. This finding is in agreement with the corresponding poor cross-immunoreactivity of serum antibodies between *Salmonella* Typhimurium and *Salmonella* Choleraesuis. Furthermore, the recombinant vaccines SLT17 (pCZ1) and SLT18 (pCZ1) resulted in 83% and 50% heterologous protection against lethal challenge with *Salmonella* Choleraesuis, respectively. The differences in the protection efficacy was correlated with differences in immunogenicity: SLT17 (pCZ1) immunization stimulated higher serum IgG and fecal IgA responses against the heterologous O-antigen and resulted in better killing of *Salmonella* Choleraesuis than SLT18 (pCZ1). We speculate that the higher amount of heterologous O-antigen expressed by SLT17 (pCZ1) than SLT18 (pCZ1) (Fig. 3) resulted in the differences in immunogenicity and protective efficacy.

Because only partial heterologous protection was achieved by the developed recombinant vaccines, considerable efforts should be made to improve the protective efficacy in future studies. One strategy is to increase the expression level of heterologous O-antigen, which might be achieved by constructing a new recombinant plasmid with a higher copy number, such as a pBR322-derived plasmid, or inserting the *Salmonella* Choleraesuis O-antigen gene cluster into a bacterial chromosome to replace the *Salmonella* Typhimurium O-antigen gene cluster, resulting in stable expression of heterologous O-antigen initiated by the native promoter^39, 44^. The other strategy is the construction of more effective recombinant *Salmonella* vectors via well-developed regulated delayed attenuation^45^. Importantly, our study provides a new approach: heterologous expression of O-antigen to develop multivalent *Salmonella* vaccines. Some issues remain to be addressed in subsequent studies, and these include whether *Salmonella* can simultaneously express two or more types of heterologous O-antigens, and ensuring the expression of sufficient amounts of heterologous O-antigens to stimulate a protective immune response when multiple O-antigens are expressed at the same time. Nevertheless, a mixture of several recombinant *Salmonella* strains, each of which express only one type of heterologous O-antigen, can be used as multivalent vaccine candidates and subjected to evaluations of immunogenicity and heterologous protection efficacy in future studies.

In summary, we developed two bivalent vaccines, SLT17 (pCZ1) and SLT18 (pCZ1), by stable recombinant expression of the heterologous *Salmonella* Choleraesuis O-antigen in attenuated *Salmonella* Typhimurium. Expression of the heterologous O-antigen had no adverse effects on bacterial swimming, resistance to polymyxin B and DOC or colonization. Immunization with SLT17 (pCZ1) and SLT18 (pCZ1) provided full protection against *Salmonella* Typhimurium infection and exhibited improved cross-protection against *Salmonella* Choleraesuis infection. The ability of the two vaccines to induce strong antibody-mediated immunity represents a promising step towards the development of live multivalent vaccines with broad serovar coverage.

## Methods

### Ethics statement

All animal experiments in this study were performed in strict accordance with the recommendations in the Guide for the Care and Use of Laboratory Animals of the Ministry of Science and Technology of China. All animal procedures were approved by the Animal Ethics Committee of the Sichuan Agricultural University.

### Bacterial strains, plasmids and growth conditions

The bacterial strains and plasmids utilized in this study are described in Table 1. *Salmonella enterica* and *E. coli* were grown in Luria-Bertani (LB) broth or on LB agar with or without 0.1% arabinose. When required, antibiotics and diaminopimelic acid (DAP) were added to the medium at the following concentrations: kanamycin, 50 μg/ml; ampicillin, 100 μg/ml; chloramphenicol, 25 μg/ml; DAP, 50 μg/ml. LB agar containing 10% sucrose was used for *sacB* gene-based counter selection in the allelic exchange experiments. The transformation of *Salmonella* Typhimurium was performed via electroporation. Transformants were selected on LB agar plates containing the appropriate antibiotics.

**Table 1:** Bacterial strains and plasmids used in this study.

Strains or plasmids	Description	Source
**Plasmids**		
pQK664	Derived from pYA3337, Asd^+^ Ptrc pSC101 origin, kan^r^	33
pSS246	pYA3700-*rfbB*	36
pCZ1	Asd^+^, pSC101 origin, Ptrc-O-antigen gene cluster of *Salmonella* Choleraesuis	This work
pYA4278	pRE112 derivative, sacB mobRP4 R6K ori Cm^r^	31
pCZ2	pYA4278-Δ*asd*	This work
pCZ3	pYA4278-Δ*rfbP*	This work
pCZ4	pYA4278-Δ*rmlB*-*rfbP*	This work
pCZ5	pYA4278-Δ*pagL*	This work
pCZ6	pYA4278-Δ*crp*	This work
pCZ7	pYA4278-Δ*cya*	This work
pCZ8	pCZ5-TT*araC* P_BAD_ *rfbP*	This work
pCZ10	pSS246-*rfbP*	This work
**Strains**		
S100	Wild-type *Salmonella* Typhimurium	51
SLT10	S100 Δ*asd38*	This work
S340	*Salmonella* Choleraesuis, wild-type virulent	36
SLT11	SLT10 Δ*rfbP39*	This work
SLT12	SLT10 Δ(*rmlB*-*rfbP*)*24*	This work
SLT13	SLT10 Δ*rfbP39* ∆*pagL32*	This work
SLT16	SLT10 Δ*rfbP39* ∆*pagL32*::TT *araC* P_BAD_ *rfbP*	This work
SLT17	SLT12 Δ*crp33* Δ*cya33*	This work
SLT18	SLT16 Δ*crp33* Δ*cya33*	This work

### Plasmids and mutant strain construction

The primers used to delete the *rfbP* gene have been described previously^31^. Other primers used in this study are listed in Supplementary Table S2. The introduction of gene mutations in *Salmonella* Typhimurium was carried out by allelic exchange using the suicide T-vector pYA4278 as previously described^31^. For deletion of the *asd* gene, the primers D*asd*-1F/D*asd*-1R and D*asd*-2F/D*asd*-2R were used to amplify approximately 450 bp upstream and downstream segments from the S100 genome, respectively. The two fragments were then joined by PCR using primers D*asd*-1F and D*asd*-2R. The PCR product was then ligated to *Ahd*I-digested pYA4278 to generate plasmid pCZ2, which was introduced into *Salmonella* Typhimurium S100 for the *asd* deletion mutation. The selection and characterization of the *asd* mutants were carried out by PCR using the primers D*asd*-1F and D*asd*-2R. The same method was applied to delete the *rfbP*, *pagL, crp* and *cya* genes and the whole O-antigen gene cluster (*rmlB*-*rfbP*). To construct the arabinose-regulated mutants, the primers *rfbP*-F and *rfbP*-R were used to amplify the *rfbP* gene from the S100 genome, and the PCR product was then inserted into plasmid pSS245^36^ between the *Nhe*I and *Kpn*I sites, generating the pCZ10 plasmid. The primers TT-F and *rfbP*-R were then used to amplify the TT*araC*P_BAD_ *rfbP* fragment from pCZ10. The product was inserted into *Not*I and *Sbf*I double-digested pCZ5 (pYA4278-Δ*pagL*), generating the plasmid pCZ8, which was introduced into the strain SLT13 (Δ*asd* Δ*rfbP* ∆*pagL*) for TT*araC*P_BAD_ *rfbP* insertion in the *pagL* gene site, generating the strain SLT16 (Δ*asd* Δ*rfbP* ∆*pagL*::TT *araC* P_BAD_ *rfbP*).

To express the heterologous O-antigen in *Salmonella* Typhimurium mutant strains, the recombinant plasmid pCZ1 was constructed. The primer pair C1-O-antigen-F and C1-O-antigen-R was used to amplify the whole O-antigen gene cluster (8870 bp) of *Salmonella* Choleraesuis from the S340 genome, and the primers pQK664-C1F and pQK664-C1R were used to amplify a DNA fragment containing the pSC101 origin, *asd* gene cassette, TIT2 terminator, Ptrc promoter and kanamycin-resistance cassette from the plasmid pQK664^33^. Next, the two PCR products were joined by the Gibson Assembly Kit (NEB, Beverley, MA, USA), generating the plasmid pCZ1. Then, the pCZ1 or the control plasmid pQK664 was transformed into *Salmonella* Typhimurium SLT11 (Δ*asd* Δ*rfbP*), SLT12 (Δ*asd* Δ*rmlB*-*rfbP*) and SLT16 (Δ*asd* Δ*rfbP* ∆*pagL*::TT *araC*P_BAD_ *rfbP*), generating the three recombinant strains SLT11 (pCZ1), SLT12 (pCZ1) and SLT16 (pCZ1) and the three control strains SLT11 (pQK664), SLT12 (pQK664) and SLT16 (pQK664).

### Sodium dodecyl sulfate-polyacrylamide gel electrophoresis (SDS-PAGE), silver staining and Western immunoblotting

To confirm the expression of *Salmonella* Typhimurium and *Salmonella* Choleraesuis O-antigens, SDS-PAGE, silver staining and Western blot analyses were performed as previously described^46, 47^. In brief, each bacterial strain was cultured and adjusted to an OD_600_ = 1.0. Two microliters of each suspension were then centrifuged and resuspended in 100 ml of lysis buffer containing proteinase K. Ten microliters of the solution were then separated by SDS-PAGE in a 12% (w/v) acrylamide gel and then silver-stained or subjected to Western immunoblotting with *Salmonella* O:4- or O:7-specific antisera diluted to 1:200 (Tianjin Biochip Corporation, Tianjin, China).

### Detection of bacterial biological activities

The bacteria were grown to an OD_600_ = 0.8–0.9 in LB broth with or without 0.1% arabinose, harvested and resuspended in LB. For the swimming assay, 6 μl of a bacterial suspension (approximately 1 × 10^6^ CFU) was spotted onto the middle of LB plates containing 0.3% agar with or without arabinose. The diameter of the colonies was measured 6 h after incubation at 37 °C. To measure the resistance to polymyxin B and DOC, 100 μl of the cell suspension (approximately 1 × 10^6^–5 × 10^6^ CFU) was inoculated with or without polymyxin B at a final concentration of 0.12 μg/ml or DOC at a final concentration of 4 mg/ml for 1 h at 37 °C. The bacteria were diluted to the appropriate concentration and plated onto LB plates. The survival rate was calculated as the CFU per ml of the polymyxin B- or DOC-treated group divided by the CFU of the non-treated group.

### Determination of bacterial colonization and virulence (LD_50_) in mice

Six-week-old female BALB/c mice were obtained from Dashuo Experimental Animal Ltd. (Chengdu, China). The mice were acclimated for 7 days after arrival before the experiments were initiated. The colonization and the LD_50_ of *Salmonella* Typhimurium strains were measured as previously described^31^. In brief, for the colonization experiments, BALB/c mice were inoculated orally with 1 × 10^9^ CFU of each strain. Six days after oral inoculation, tissues, including PP, spleen and liver, were collected from three animals, weighed and homogenized in 1 ml of PBS. Then, appropriate dilutions were plated onto MacConkey agar or LB agar to determine the number of viable bacteria. The colonization value was calculated as CFU per gram of tissue (CFU/g). For determination of the LD_50_, stepwise increasing doses of *Salmonella* Typhimurium strains were orally inoculated into groups of BALB/c mice. The mortality of the animals was monitored over a period of 1 month after infection. The LD_50_ was calculated using the method of Reed and Muench.

### Immunization and challenge

The *Salmonella* vaccine strains SLT17 (pQK664), SLT17 (pCZ1), SLT18 (pQK664) and SLT18 (pCZ1) were grown statically overnight in LB broth (0.1% arabinose was added for culture of the arabinose-regulated vaccine strains) at 37 °C. The next day, the overnight culture was diluted 1:100 into 100 ml of LB broth (0.1% arabinose was added for culture of the arabinose-regulated vaccine strains) and grown with shaking at 37 °C to an OD_600_ value of 0.8 to 0.9. The cells were harvested by centrifugation and resuspended in 1 ml of phosphate buffer saline (PBS). Groups of mice (12 mice per group) were orally inoculated with 20 µl of PBS containing 1 × 10^9^ CFU of each strain or with PBS without bacteria twice at an interval of 4 weeks. The serum was collected three weeks after each immunization. The feces used for the analysis of fecal IgA were collected three weeks after the second immunization and suspended in 100 µl of PBS, and the supernatant was collected after centrifugation at 12,000 × g and 4 °C for 5 min. One month post-second immunization, mice in each immunization group were orally challenged with at least 100 × LD_50_ of the *Salmonella* Typhimurium virulent strain S100 and the *Salmonella* Choleraesuis virulent strain S340, respectively. The challenged mice were monitored, and mortality was recorded daily for 15 days.

### ELISA

*Salmonella* Typhimurium LPS was purchased from Sigma (St. Louis, MO, USA), and *Salmonella* Choleraesuis LPS was extracted and purified as described^48^. The serum IgG concentration was determined by quantitative ELISA, and the fecal IgA was measured by indirect ELISA, as previously described^36, 49^. In brief, a 96-well ELISA microplate was coated with 100 ng/well LPS or 100 ng/well goat anti-mouse Ig(H + L) (BD, San Diego, CA, USA) and then blocked with 2% BSA (BD) after overnight incubation at 4 °C. For the detection of serum IgG, the serum samples were diluted to 1:100 in PBS containing 2% BSA, and 100 μl of this solution was added to the LPS-coated wells in duplicate. Meanwhile, 100 μl of mouse IgG (BD) with serial two-fold dilutions from 0.5 mg/ml were added to the goat anti-mouse Ig(H + L)-coated wells for the standard curve. For the detection of fecal IgA, the fecal supernatant was diluted 1:4 and added to the LPS-coated wells in duplicate. After 1 h of incubation at 37 °C, 100 μl of biotinylated goat anti-mouse IgG (Southern Biotech, Birmingham, AL, USA) or biotinylated goat anti-mouse IgA (Southern Biotech) and 100 μl of streptavidin-alkaline phosphatase conjugate (Southern Biotech) were added to each well sequentially, followed by p-nitrophenylphosphate substrate in diethanolamine buffer. Finally, the plate was read at 405 nm using a microplate reader (Bio-Rad Laboratories, California, USA). The standard curve was drawn using Curve Expert (Hyams DG, Starkville, MS, USA), and the concentration of serum IgG antibodies was calculated according to the standard curve.

### Serum bactericidal assay

The serumcollected from mice of each immunized group at week 3 post-second immunization was pooled for the serum bactericidal assay as described previously^50^. The commercial *Salmonella* O:4-specific and O:7-specific antisera were used as positive controls. All serum samples were heated at 56 °C for 30 min to inactivate endogenous complement. *Salmonella* Typhimurium S100 or *Salmonella* Choleraesuis S340 were grown in LB medium to log phase and were diluted in SBA buffer (50 mM phosphate, 0.041% MgCl_2_·6H_2_O, 33 mg/ml CaCl_2_, 0.5% BSA) to approximately 3 × 10^3^ CFU/ml. The bacteria were incubated with 25% heat-inactivated serum supplemented with or without active guinea pig complement (Sigma) for 1.5 h. The relative survival was calculated as the percent CFU counted in each pooled serum with active complement compared to the CFU of the same serum with no complement. Each sample and control was tested in triplicate.

### Statistical analysis

The data are shown as the means ± SD. One-way ANOVA followed by Tukey’s multiple-comparison test were used to evaluate statistical significance. A probability value of *P* < 0.05 was considered statistically significant. The survival curve post-challenge was analyzed using the log-rank test. All *in vitro* experiments were repeated at least three times, and the *in vivo* experiments were repeated at least twice.

### Data Availability

The datasets generated during and/or analysed during the current study are available from the corresponding author on reasonable request.

## Electronic supplementary material

Supplementary Information
