# Supplementary material for: Recombinant attenuated Salmonella Typhimurium with heterologous expression of the Salmonella Choleraesuis O-polysaccharide: high immunogenicity and protection
Source: Sci Rep. 2017 Jul 28;7:7127. doi: 10.1038/s41598-017-07689-5 (PMC5533773; doi:10.1038/s41598-017-07689-5)
Supplement: Supplementary file 1 — Supplementary Information [file 41598_2017_7689_MOESM1_ESM.pdf]

Recombinant attenuated *Salmonella* Typhimurium with heterologous expression of the *Salmonella* Choleraesuis O-polysaccharide: high immunogenicity and protection

Xinxin Zhao, Qinlong Dai, Dekang Zhu, Mafeng Liu, Shun Chen, Kunfeng Sun, Qiao Yang, Ying Wu, Qingke Kong, Renyong Jia

Supplementary Information

Supplementary Table S1. The virulence of wild-type *Salmonella* strains and the recombinant vaccines.

Strains	LD_{50} (CFU)
S100	5.4×10^5
S340	2.9×10^5
SLT17 (pQK664)	$> 2\times10^9$
SLT17 (pCZ1)	$> 6\times10^9$
SLT18 (pQK664)	$> 8\times10^9$
SLT18 (pCZ1)	$> 5\times10^9$

Supplementary Table S2. Primers used in this study.

Primer	Sequence 5'-3'
D*asd*-1F	CGGCGCGATTGTCGGGATG
D*asd*-1R	CGCCCCATAAAGCGTTTTTTTCCTGCAAAG
D*asd*-2F	GGAAAAAAACGCTTTATGGGGCGCCGC
D*asd*-2R	GTCCGGCTTGGGTCTGGTGC
D*pagL*-1F	TGCGGATGAAGCTGCCGACC
D*pagL*-1R	CCTGCAGGATGCGGCCGCTGAAGTTGAATAACAATTAGCG
D*pagL*-2F	GCGGCCGCATCCTGCAGGCTCCACCACCATTTCAATGTC
D*pagL*-2R	AGACTATCTTTACTGGCAGG
rfbP-F	CTAGCTAGCGGAAGTCATTATGGATAATATTGATAATAAGTATAATCC
rfbP-R	CGGGGTACCCTGCAGGTTAATACGCACCATCTCGCC
TT-F	ATGCGGCCGCAGATCTTTTATTATTCTATCC
D*rmlB*-*rfbP*-1F	GTTGGCTGAAACGAGTGTTG
D*rmlB*-*rfbP*-1R	GGTAAGCGCGGCCGCTTTTCTATTCCATAAGGCGT
D*rmlB*-*rfbP*-2F	TAGAAAAGCGGCCGCGCTTACCGAGAAGTACTGAAT
D*rmlB*-*rfbP*-2R	GGCACCAGCTTTTTGCCGGG
C1 O-antigen-F	CACACAGGAAACAGAATGATATATTATATTTTTATTGTAATTTTTCCG
C1 O-antigen-R	TCCGCCAAAACAGCCGGTATAACCACGGCTTTCGATGTTGAGC
pQK664-C1F	AGCCGTGGTTATACCGGCTGTTTTGGCGGATGAGAGAAGATTTTC
pQK664-C1R	AATATAATATATCATTCTGTTTCCTGTGTGAAATTGTTATCC
D*crp*-1F	CGCAGTTGGTGACATTCTGACG
D*crp*-1R	TCTGACGGAAGCGCGGTTATCCTCTG
D*crp*-2F	ATAACCGCGCTTCCGTCAGAATGGCGC
D*crp*-2R	GCCATAGCCAGAACCAAAACCA
D*cya*-1F	GGATGGGTTGCCATCAATGC
D*cya*-1R	CGAAAAATACTGACGTATCGCCTGATGTTGC
D*cya*-2F	GGCGATACGTCAGTATTTTTCGTAAGTGCTGCG
D*cya*-2R	CGGCACAGTATCGCTCTAAAG

Figures and Figure legends

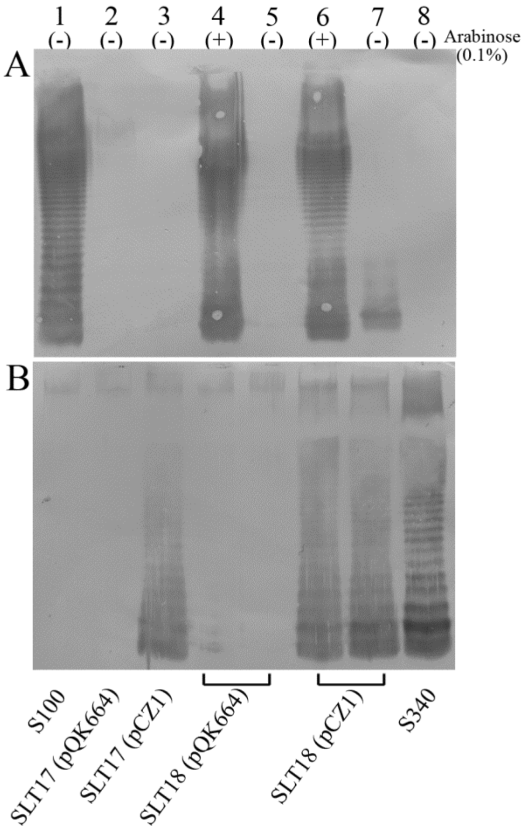

Supplementary Figure S1. Detection of LPS phenotypes of the vaccine strains by Western immunoblotting. LPS extracted from *Salmonella* Typhimurium wild-type S100 (lane 1), SLT17 (pQK664) (lane 2), SLT17 (pCZ1) (lane 3), SLT18 (pQK664) grown with arabinose (lane 4) or without arabinose (lane 5), SLT18 (pCZ1) grown with arabinose (lane 6) or without arabinose (lane 7) and *Salmonella* Choleraesuis S340 (lane 8) was subjected to SDS-PAGE followed by immunoblotting using O:4-specific antisera (A) and O:7-specific antisera (B).

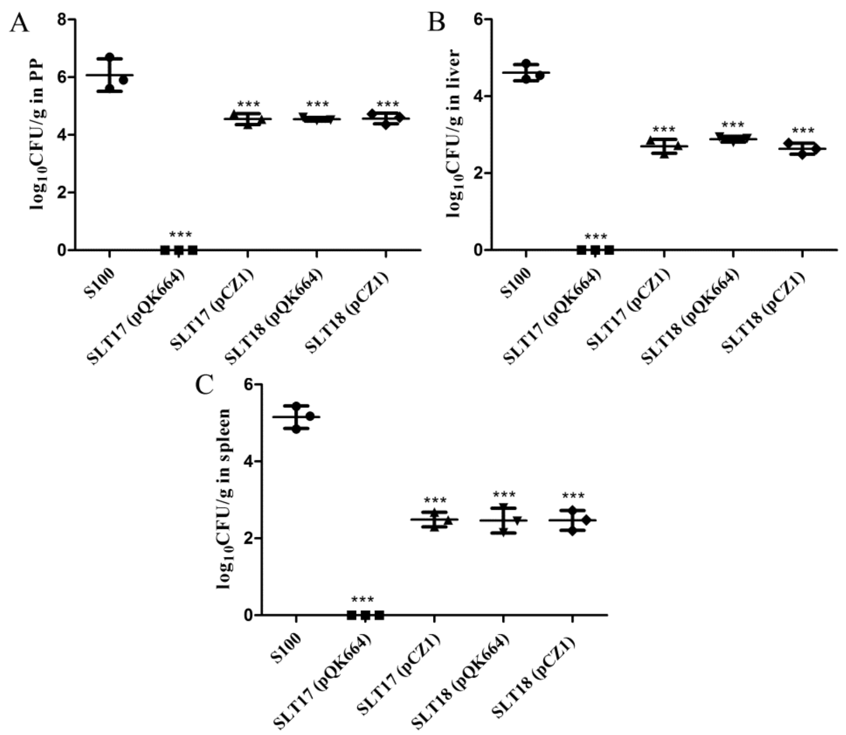

Supplementary Figure S2. Colonization of mice by *Salmonella* Typhimurium wild-type S100 and four vaccine strains. Groups of BALB/c mice (4 mice/group) were orally inoculated with approximately 1×10^9 CFU of each indicated strain. Viable bacteria were recovered from PP (A), liver (B) and spleen (C) 6 days after infection. The bacterial number in each tissue was calculated as $\log_{10}$CFU/g. The asterisk above the error bar indicates significance compared to the S100 group. ***, p<0.001.
